# The association between childhood trauma and overweight and obesity in young adults: the mediating role of food addiction

**DOI:** 10.1007/s40519-022-01454-y

**Published:** 2022-07-30

**Authors:** Samuel Offer, Elise Alexander, Kelsie Barbara, Erik Hemmingsson, Stuart W. Flint, Blake J. Lawrence

**Affiliations:** 1 Discipline of Psychology, School of Population Health, Curtin University, Perth, WA Australia; 2 Medical School, Curtin University, Perth, WA Australia; 3 Swedish School of Sport and Health Sciences, Stockholm, Sweden; 4 School of Psychology, University of Leeds, Leeds, UK; 5 Scaled Insights, University of Leeds, NexusLeeds, UK

**Keywords:** Childhood trauma, Food addiction, Overweight, Obesity, Young adults

## Abstract

**Purpose:**

Childhood trauma is associated with increased risk of obesity during adulthood, which may be associated with the development of food addiction. This study examined whether food addiction mediated the relationship between childhood trauma and obesity in young adults.

**Methods:**

A sample of 512 young adults, aged 18 to 30 years, living with overweight and obesity (Body Mass Index ≥ 25 kg/m^2^), from the United Kingdom participated in the study. Participants completed the Childhood Trauma Questionnaire (CTQ), the Yale Food Addiction Scale, and provided their current height and weight to compute their Body Mass Index (BMI).

**Results:**

Using the PROCESS macro, a mediation analysis found that food addiction accounted for 45% of variance in the relationship between childhood trauma and BMI. Post hoc analyses were conducted to examine the mediating effect of food addiction across each of the five subscales of the CTQ (emotional/physical/sexual abuse and emotional/physical neglect). Food addiction accounted for 32% to 51% of the variance in the relationship between each CTQ subscale and BMI.

**Conclusions:**

These findings suggest that experiences of childhood trauma are associated with the development of overweight and obesity during early adulthood and up to half of this relationship can be attributed to food addiction, which is likely used as a maladaptive coping mechanism in response to trauma. Young adults living with overweight and obesity who report experiences of childhood trauma may benefit from the support of clinical and counselling psychologists to improve their understanding of the underlying psychosocial factors that influence their eating behaviours.

**Level of evidence:**

Level V, cross-sectional analytic study.

## Introduction

Approximately 1.9 billion adults globally are currently living with overweight and 650 million with obesity [1]. Much of the literature examining the incidence of obesity has focused on lifestyle factors, including a poor diet and a lack of physical activity, as the leading causes of weight gain [2]. However, interventions which focus on lifestyle factors alone are often unsuccessful long-term, with evidence indicating that approximately 80% of people living with overweight do not maintain a weight loss of ≥ 10% of total body weight for more than a year [3]. A meta-analysis of 45 studies examining the effectiveness of diet and exercise interventions found that the implementation of these interventions only had a small effect on long-term weight loss in adults [4]. These findings suggest that lifestyle only interventions are unlikely to lead to sustained weight loss among people living with overweight or obesity. Other evidence suggests that socioeconomic adversity and psychological experiences may also contribute to the development of overweight and obesity [5], but further research is needed to understand their impact on weight gain from childhood through to adolescence and adulthood. Obesity appears to be a complex, multifactorial health condition, where there are wide-ranging, interconnected factors that can lead to weight gain and thus obesity, with many of these factors either outside or partially outside of an individual’s control [6]. Examples of other factors that are associated with an increase likelihood of overweight or obesity during adulthood, include extent of socioeconomic deprivation and negative psychological experiences during childhood [5].

Evidence suggests that psychosocial risk factors, such as traumatic experiences during childhood, may increase the risk of developing obesity during adulthood [7–9]. A theoretical model of obesity causation has been proposed by Hemmingsson [5], which identifies how a series of negative psychosocial events may occur during childhood/adolescence and consequently lead to the development of overweight or obesity in adulthood. For example, when a child experiences a high degree of psychological and emotional stress, this may lead to overconsumption of calorie-dense foods (i.e., junk food) as a maladaptive coping mechanism in response to the stressful life events. When left unaddressed, negative psychosocial experiences and the ongoing consumption of unhealthy food can significantly increase the risk of developing overweight and obesity. This process of weight gain is often influenced by stressors in the social environment which can lead to an increased release of hormones such as cortisol, resulting in a subsequent increase in appetite and desire for calorie-dense foods [10, 11]. Negative coping mechanisms, such as overeating, are often maintained in an attempt to cope with the continued exposure to psychosocial stressors, which often leads to greater weight gain and also increases the risk of bullying, low self-esteem, and the development of psychological disorders, which can be exacerbated by the presence of weight stigma [12]. Therefore, each stage of this model has the potential to reinforce a previous stage in a negative feedback loop, which can have a significant physiological and psychological impact on an individual living with overweight or obesity [5]. Recent research has identified that different forms of childhood trauma (i.e., emotional, physical, sexual) may be primary risk factors that contribute to the development of overweight and obesity during adolescence and early adulthood [9].

Childhood trauma can have a detrimental impact on the psychological and physiological development of children and adolescents [13]**.** For instance, evidence shows that childhood trauma can increase the risk of depression, anxiety, post-traumatic stress disorder [14] and eating disorders [15]. In addition, childhood trauma can significantly affect brain development and function, including areas of the brain associated with the regulation of stress [16]. Due to this reduced capacity to manage stress, individuals who have experienced childhood trauma may be at an increased risk of substance abuse disorders, including the use of alcohol and illicit drugs to cope with negative emotions [17]. Other coping strategies to manage negative emotions, such as overeating, are also more common in those who have experienced childhood trauma, which can increase the likelihood of developing obesity in adulthood [13, 18]. Several extensive meta-analyses show that individuals are significantly more likely to develop obesity during adulthood if they have previously experienced childhood trauma [7–9, 19]. However, while the evidence suggests that childhood trauma is associated with obesity, the underlying mechanisms involved in this relationship are not yet known.

Food addiction may contribute to the relationship between childhood trauma and the development of obesity during early adulthood [20]. Food addiction is described as the inability to control the consumption of favourable foods, particularly processed foods containing additional sugars and fats (i.e., junk food), through mechanisms similar to those which underlie substance addiction [21]. Emerging research has shown that several biological and behavioural changes occur in those with food addiction, such as differences in brain reward processes, which result in impaired control and the development of tolerance and withdrawal symptoms [22]. These notable changes which occur in those with food addiction are similar to those found in individuals with substance abuse/addiction disorders [23], suggesting that similar mechanisms may be involved in the development and maintenance of food addiction [23]. A primary consequence of food addiction is weight gain and evidence from a large meta-analysis (*n* = 196,211) shows that compared to adults living with a healthy weight, adults living with overweight or obesity are more than twice as likely to report symptoms of food addiction [24]. While the development of overweight or obesity may be an obvious consequence of food addiction, the relationship between food addiction, childhood trauma, and overweight or obesity is less clear.

Few studies have examined the relationship between childhood trauma and food addiction. A preliminary study showed that food addiction may be associated with several subdomains of childhood trauma, including emotional, physical, and sexual abuse, as well as emotional and physical neglect [21]. The study sampled 231 adults and found small to moderate effect sizes between these subscales of childhood trauma and food addiction [21]. However, the study recruited outpatients from an eating disorders clinic, which may limit the external validity of these findings. Similarly, another study found that an increased severity of childhood trauma was correlated with a significantly higher risk of developing food addiction in a sample of female nurses; severe childhood trauma was associated with an approximately 90% increase in food addiction risk [25]. A different study found that those who had experienced childhood trauma were more likely to have severe food addiction symptoms, in a group of participants who were seeking bariatric surgery [26]. However, the external validity of these studies was also limited, as the samples recruited were not representative of the general population [25, 26].

While these studies report methodological limitations regarding recruitment, a recent study has demonstrated the relationship between food addiction and childhood trauma in a non-clinical sample [20]. The study recruited 186 participants (84 males and 102 females) who did not have any diagnosed psychiatric or medical conditions, reporting that in participants with high BMI (> 25 kg/m^2^), food addiction was significantly correlated with early life adversity. This relationship between food addiction and childhood trauma, may explain the association between childhood trauma and obesity, as food addiction is significantly associated with higher BMI scores [24]. However, no study to date has examined the role of food addiction in the relationship between childhood trauma and obesity development. Therefore, the aim of this study is to explore the potential mediating role of food addiction in this relationship. The following hypotheses are proposed:

- *H1:* Childhood trauma will be significantly and positively associated with BMI.
- *H2:* Childhood trauma will be significantly and positively associated with food addiction.
- *H3:* Food addiction will be significantly and positively correlated with BMI.
- *H4:* Food addiction symptoms will significantly mediate the relationship between childhood trauma and BMI.

## Method

### Research design and participants

This study used a cross-sectional research design using an online questionnaire developed in Qualtrics™, and participants were recruited using Prolific Survey Pooling Service (prolific.co). The data were collected on the 9th of July, 2021. The participant inclusion/exclusion criteria were pre-determined through the Prolific platform prior to beginning data collection. Participants were excluded from the study if they were not currently living with overweight or obesity (BMI < 25 kg/m^2^), did not reside in the United Kingdom, and were either < 18 years or > 30 years. A Monte Carlo power analysis was used to determine the sufficient sample size for this study [27]. Following 10,000 replications, to achieve a desired power of 0.80 at *α* = 0.05, it was determined that 465 participants would be required for the mediation analysis to be sufficiently powered to detect small effects (*β* = 0.15), as recent studies have found small effect sizes [21]. To account for potential participant attrition, 595 participants were recruited for this study.

## Measurements

### Childhood trauma questionnaire—short form

The Childhood Trauma Questionnaire—Short Form (CTQ-SF) was used to measure childhood trauma [28]. This measure includes 25 items across 5 subscales of childhood trauma, including emotional, physical, and sexual abuse, as well as emotional and physical neglect. A high score on the CTQ-SF indicates that the participant has experienced a severe level of childhood trauma, with scores ranging from 25–125. The full-length CTQ scale has demonstrated internal consistency in a community sample (Cronbach’s alpha = 0.91) [29] as well as test–retest reliability in a range of samples, with coefficients ranging from 0.79 to 0.86 over an average of four months [30]. It has a consistent five-factor structure across a range of samples, indicating the suitability of the childhood trauma subscales [30]. The subscales of the CTQ have demonstrated convergent validity with corresponding subscales on the Child Maltreatment Ascertainment Interview and discriminant validity with unrelated subscales from the same measure [28].

### Yale Food Addication Scale 2.0

The second version of the Yale Food Addiction Scale (YFAS 2.0) was used to measure food addiction symptoms [31]. This measure includes 35 items and collects participants responses to symptoms of food addiction using an eight-point Likert scale, ranging from “0 = never” to “7 = every day”. From these responses, a total symptom count of food addiction can be calculated, with symptom counts ranging from 0–11. This scale has demonstrated internal consistency in a community sample (Cronbach’s alpha = 0.92) as well as a consistent one-factor structure [31]. The YFAS 2.0 has demonstrated convergent validity with the disinhibition and hunger subscales from the Three Factor Eating Questionnaire, as well as discriminant validity with dietary restraint from the same measure [31].

### Body Mass Index and demographic information

Participant height and weight was collected to calculate body mass index (BMI) for each participant. BMI is calculated by dividing a participant’s weight (in kilograms) by the square of their height (in meters). According to the World Health Organization, individuals with a BMI of 25 kg/m2 to 29.9 kg/m2 are considered as living with overweight, while individuals with a BMI of 30 kg/m2 or more as considered as living with obesity. Demographic information, including the participants’ age, gender, ethnicity, and relationship status were also collected.

### Procedure

This study was approved by Curtin University’s Human Research Ethics Committee (HRE2021-0340) on the 15th of June, 2021. The questionnaire was uploaded via the Prolific platform and participants who met the eligibility criteria were invited to participate in the study. The first page of the questionnaire provided participants an information sheet which included instructions, as well as risks and benefits involved with participation in the study. Participants indicated their informed consent by checking a box prior to completing the questionnaire. The questionnaire took approximately 12 min to complete. Following the completion of the questionnaire, participants were thanked for their time and provided with the lead researcher’s contact information for follow-up questions. As some of the questions were of a sensitive nature, the contact numbers of several primary UK mental health services were provided. Participants were reimbursed £1.50 (approximately $2.70 AUD) for their time, following the completion of the questionnaire, which is the recommended reimbursement rate for a 12-min questionnaire on the prolific recruitment platform.

## Results

Following data cleaning and screening, the final sample size included 514 young adults (age *M* = 25.39, *SD* = 3.57) living with overweight or obesity. Participant descriptive statistics are reported in Table 1 and Pearson correlation coefficients are reported in Table 2. As shown by Table 1, the sample of this study involved largely female participants who were of white ethnicity. Table 2 shows that childhood trauma, food addiction, and BMI were statistically significantly and positively correlated, and that the strength of these associations were considered weak.

**Table 1:** Descriptive statistics of participants (*N* = 514)

	BMI		YFAS 2.0		CTQ-SF	
Characteristic	*M*	*SD*	*M*	*SD*	*M*	*SD*
Total (*N* = 514)	37.95	10.62	5	3.77	45.42	18.77
Gender						
Female (*N* = 367)	38.37	10.12	5.22	3.83	45.34	21.27
Male (*N* = 130)	35.31	8.98	4.33	3.52	43	16.22
Non-binary/third gender (*N* = 17)	49.09	20.66	5.41	3.65	63.77	21.27
Marital status						
Single (*N* = 235)	37.59	11.24	5.07	3.67	46.15	17.27
Partner (*N* = 198)	37.34	9.49	4.87	3.86	44.96	19.70
Married (*N* = 69)	40.69	11.66	5.19	3.90	45.12	21.58
De Facto (*N* = 10)	39.98	7.13	5.20	3.52	38.50	12.31
Saparated (*N* = 1)	43.42	–	8	–	26	–
Divorced (*N* = 1)	27.05	–	0	–	47	–
Ethnicity						
White (*N* = 430)	38.29	10.90	4.96	3.78	44.44	18.32
Black/African/Caribbean (*N* = 19)	34.70	6.21	4.90	3.91	54.52	20.41
Asian (*N* = 42)	36.18	8.93	5.52	3.66	50.27	21.28
Mixed/Multy ethnic group (*N* = 20)	37.18	11.33	4.65	3.73	45.40	17.38
Other ethnic group (*N* = 3)	39.87	3.47	7	3.61	50.33	26.63

^*^p < 0.05; ^**^p < 0.01; ^***^p < 0.001

**Table 2:** Correlation matrix of study variables

	1	2	3
1. Age	–		
2. BMI	0.13**	–	
3. YFAS 2.0 (symptom score)	− 0.01	0.33***	–
4. CTQ-SF (total score)	− 0.09*	0.20***	0.31***

^*^p < 0.05; ^**^p < 0.01; ^***^p < 0.001

**Fig. 1:**
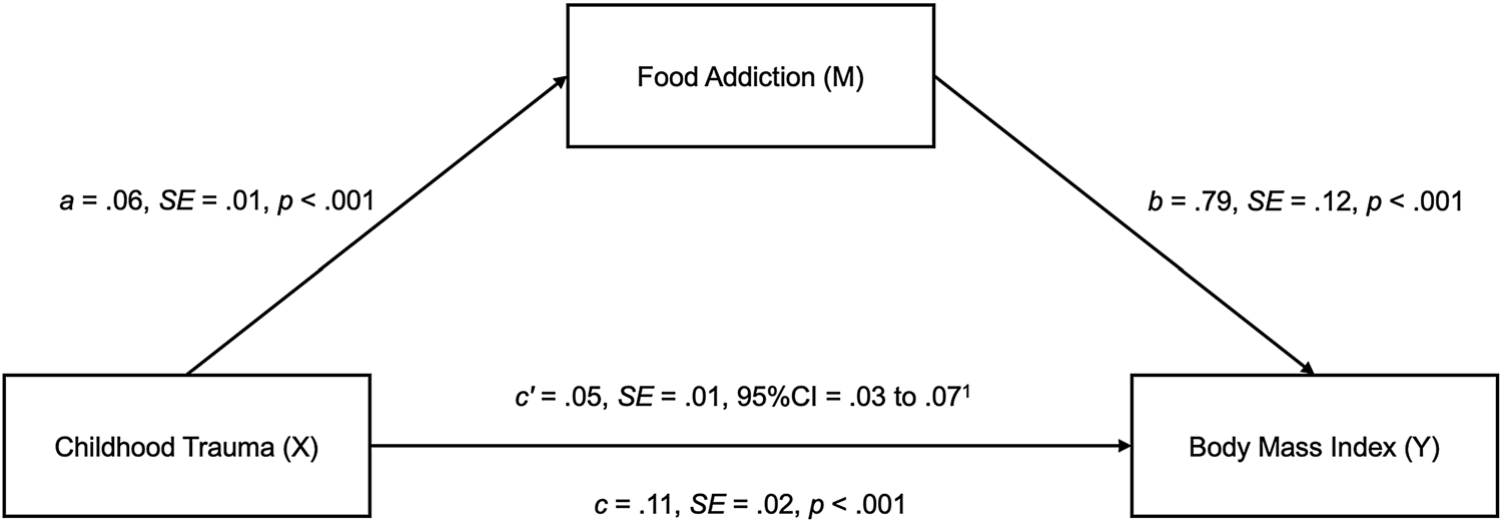
The mediating effect of food addiction in the relationship between childhood trauma and BMI. Note ^1^ = The PROCESS macro does not produce P values for the indirect effects, but the confidence interval does not include zero which indicates presence of statistical significance

To determine whether food addiction mediated the relationship between childhood trauma and BMI, a mediation analysis was conducted using the PROCESS macro in SPSS (version 27). Age was significantly associated with BMI and there was a statistically significant difference (*p* < 0.05) in BMI across gender classifications (male, female, non-binary/third gender), therefore age and gender were controlled in the mediation model as covariates. Following 10,000 bootstrapped samples, childhood trauma was positively associated with food addiction (path *a*), food addiction was positively associated with BMI (path *b*), and childhood trauma was positively associated with BMI (path *c*). The indirect effect of childhood trauma to BMI, through food addiction, was significant (path *c*’), with food addiction accounting for a significant 45.5% of the variance between childhood trauma and BMI in this sample of young adults living with overweight or obesity (refer to Fig. 1). Therefore, *H4* was also supported.

With food addiction accounting for a statistically significant proportion of variance in the relationship between childhood trauma (as measured by the total score from the CTQ-SF) and BMI, exploratory post hoc analyses were conducted to examine whether food addiction mediated the relationships between each of the subscales from the CTQ-SF and BMI in this sample of young adults. Given that these additional analyses were exploratory, a Bonferroni correction for multiple comparisons was not applied to the alpha level for determining statistical significance. While controlling for age and gender, food addiction accounted for a statistically significant 51.5% of the variance between emotional abuse and BMI, 45.2% of the variance between physical abuse and BMI, 32.6% of the variance between sexual abuse and BMI, 49.2% of the variance between emotional neglect and BMI, and 49.9% of the variance between physical neglect and BMI. Please see Table 3 for the full results from these post hoc mediation analyses.

**Table 3:** Mediating effects of food addiction on each subscale of the Childhood Trauma Questionnaire—Short-Form

Childhood Trauma Questionnaire Subscales	Coefficient	*SE*	*p*	95% CI	Effect size (%)
Emotional Abuse Subscale					
Total effect of emotional abuse on food addiction (path *a*)	0.22	0.03	00.001***	0.17 to 0.28	
Total effect of food addiction on BMI (path *b*)	0.80	00.12	0.001***	0.56 to 1.04	
Total effect of emotional abuse on BMI (path *c*)	0.35	0.08	0.001***	0.19 to 0.50	
Indirect effect of emotional abuse on BMI, through food addiction (path *c’*)	0.18	0.04	–	0.11 to 0.26^1^	51.5
Physical Abuse Subscale					
Total effect of physical abuse on food addiction (path *a*)	0.23	0.04	0.001***	0.14 to 0.31	
Total effect of food addiction on BMI (path *b*)	0.83	12	0.001***	0.59 to 1.06	
Total effect of physical abuse on BMI (path *c*)	0.41	0.12	0.001**	0.18 to 0.65	
Indirect effect of physical abuse on BMI, through food addiction (path *c’*)	0.19	0.04	–	0.11 to 0.28^1^	45.2
Sexual Abuse Subscale					
Total effect of sexual abuse on food addiction (path *a*)	0.10	0.03	0.002**	0.04 to 0.17	
Total effect of food addiction on BMI (path *b*)	0.85	0.12	0.001***	0.62 to 1.08	
Total effect of sexual abuse on BMI (path *c*)	0.27	0.09	0.004**	0.09 to 0.46	
Indirect effect of sexual abuse on BMI, through food addiction (path *c’*)	0.09	0.03	–	0.04 to 0.15^1^	32.6
Emotional Neglect Subscale					
Total effect of emotional neglect on food addiction (path *a*)	0.19	0.03	0.001***	0.13 to 0.24	
Total effect of food addiction on BMI (path *b*)	0.82	0.12	0.001***	0.58 to 1.06	
Total effect of emotional neglect on BMI (path *c*)	0.31	0.08	0.001***	0.15 to 0.47	
Indirect effect of emotional neglect on BMI, through food addiction (path *c’*)	0.15	0.03	–	0.09 to 0.23	49.2
Physical Neglect Subscale					
Total effect of physical neglect on food addiction (path *a*)	0.21	0.04	0.001***	0.12 to 0.30	
Total effect of food addiction on BMI (path *b*)	0.85	0.12	0.001***	0.61 to 1.08	
Total effect of physical neglect on BMI (path *c*)	0.35	0.12	0.005**	0.11 to 0.60	
Indirect effect of physical neglect on BMI, through food addiction (path *c’*)	0.18	0.05	–	0.09 to 0.29	49.9

*SE* standard error; *CI* confidence interval

** = *p* < 0.01; *** = *p* < 0.001

^1^The PROCESS macro does not produce *p* values for the indirect effects, but each confidence interval does not include zero which indicates presence of statistical significance

## Discussion

The findings from this study show that experiences of childhood trauma are associated with increases in BMI in young adults living with overweight or obesity, and food addiction may explain up to 50% of this relationship. These findings support Hemmingsson’s [5] model of obesity causation which proposes how up-stream events and circumstances during childhood often lead to negative down-stream effects during adolescence and early adulthood. Specifically, individuals who experience traumatic events during childhood may turn to the consumption of food as a maladaptive coping mechanism, which over time often leads to weight gain and the onset of overweight or obesity [5]. Our findings support this model of obesity causation and provide preliminary evidence of the extent to which food addiction may affect the relationship between different subtypes of childhood trauma and BMI in young adults living with overweight and obesity.

Emotional abuse refers to verbal attacks on a child, or demeaning behaviour directed toward a child by an older person, including shaming and humiliation. Current study findings indicate that food addiction accounted for 51% of the variance in the relationship between emotional abuse and BMI. This finding is consistent with previous research, which also found an association between emotional abuse and food addiction [21]. An earlier study found similar results, with emotional abuse the strongest predictor of disordered eating habits when compared to other types of abuse [32]. In addition, a meta-analysis found that experiences of emotional abuse increased the likelihood of developing adulthood obesity by 36% [8]. Current study findings support and extend previous findings, by reporting the first evidence of how food addiction may mediate the relationship between emotional abuse and BMI in young adults. In addition to emotional abuse, food addiction also mediated the relationship between physical abuse and BMI, which supports previous findings that have reported associations between physical abuse, food addiction, and disordered eating [21]. A meta-analysis found that experiencing physical abuse during childhood increased the likelihood of developing obesity by 28% [8]. It was also found that food addiction accounted for 32% of the variance in the relationship between sexual abuse and BMI. Food addiction accounted for a significant proportion of the variance between sexual abuse and BMI, although it was the smallest proportion of any CTQ subscale. This supported previous findings, as although sexual abuse was significantly correlated with food addiction, it was the smallest correlation of any of the CTQ subscales [21]. While some studies have found sexual abuse to be significantly associated with disordered eating [33, 34], other studies have found that sexual abuse was not a significant predictor of disordered eating [32, 35, 36]. However, a meta-analysis found that sexual abuse was associated with an increased BMI, as experiences of sexual abuse increased the likelihood of developing adulthood obesity by 31% [8]. Therefore, while sexual abuse appears to elicit food addiction as a maladaptive coping mechanism, there may be other important mechanisms present in the development of obesity. These mechanisms may differ in those who have experienced other types of childhood trauma. Future research should examine the potential underlying mechanisms (i.e., mood disorders) that may differentiate between the different types of abuse.

Physical neglect refers to a child’s physical needs not being met by a caretaker, which includes providing necessities such as food and shelter. Current study findings indicate that food addiction accounted for 50% of the variance in the relationship between physical neglect and BMI, which was the second largest proportion of variance which was accounted for by food addiction out of the CTQ subscales. This is consistent with previous findings, where physical neglect was also the subscale with the second largest correlation to food addiction, behind emotional abuse [21]. While studies examining the individual role of physical and emotional neglect on eating disorders are limited, a meta-analysis found that those who had experienced overall childhood neglect were three times more likely to experience disordered eating [37]. In addition to physical neglect, emotional neglect refers to a child’s emotional and psychological needs not being met by a caretaker, which can include the need for love and support. Current study findings indicate that food addiction accounted for 48% of the variance in the relationship between emotional neglect and BMI. This is consistent with previous findings, as emotional neglect was significantly associated with food addiction [21]. While food addiction accounted for a similar percentage of variance in the relationships between physical and emotional neglect and BMI, future research should examine the potential underlying mechanisms that may differentiate between these different types of neglect.

It is important to note that completion of the CTQ requires participants to reflect on past experiences of childhood trauma. These findings may therefore indicate presence of a current emotional response to their past trauma, which is similar across all CTQ subscales used in this study. As noted above, there may be similar mechanisms associated with the development of food addiction in response to each type of childhood trauma. In addition, the CTQ subscales are highly correlated [28], which also suggests that the experience of emotional, physical, and sexual abuse as well as emotional and physical neglect, may elicit similar coping mechanisms, which supports our consistent finding of food addiction mediating the relationships between each domain of the CTQ with BMI. Future research should explore potential underlying factors across each subscale which might affect the likelihood of developing food addiction. Previous research has found that childhood trauma may be reflective of poor family functioning, which may also be an underlying factor in the development of food addiction which is non-specific to any individual domain of childhood trauma [38].

## Strengths and limits

Current study findings have provided new insights and extended the current knowledge of the mediating role of food addiction in the relationship between childhood trauma and obesity in young adults, as this relationship has not been examined in previous research. A notable strength of the present study was the sample size (*n* = 514) which provided sufficient power to explore a series of mediation models. In addition, this study included a large sample of young adults living with overweight and obesity in a Western population (i.e., United Kingdom), which permits these findings to be generalised to many other young adults living in Western countries. Approximately 64% of people living in Western nations, such as the UK, are classified as living with overweight and obesity [39]. It is therefore imperative that future research builds upon the novel findings from this study and continues to improve our understanding of the underlying psychological factors that likely predispose a large proportion of the population to a significant increased risk of developing, and most likely maintaining, overweight and obesity, and the associated health risks. There are also limitations of the present study. First, a cross-sectional design was used which required adult participants to reflect on previous experiences of childhood trauma. Adult participants may not accurately recall the extent of their childhood experiences, with evidence suggesting that experiences of childhood trauma are often underreported [40]. The findings from this study may therefore underestimate the extent of childhood trauma experienced by young adults living with overweight or obesity. In addition, the use of psychotherapies, such as Eye Movement Desensitization and Reprocessing (EMDR), can significantly influence the perception of experiences of past trauma, reducing the presence and intensity of negative emotions from the traumatic experiences [41]. Unfortunately, we did not collect this demographic data from participants, therefore it is not possible to determine from the current data what impact any previous completion of EMDR, or other similar psychotherapeutic interventions, may have had on the self-report scores from the childhood trauma questionnaire used in this study. A further limitation is that additional information regarding the participants’ psychological profile was not collected in this study. As the presence of personality disorders [42] and emotional dysregulation [43] can also influence the development of eating disorders, future studies examining the impact of eating disorders on the development of food addiction, in those who have experienced childhood trauma, are necessary. BMI is also a crude measure of overweight and obesity and cannot differentiate between an individual’s muscle mass or fat mass. Future studies examining the relationships between childhood trauma, food addiction, and overweight and obesity, will benefit from using more accurate measures of body mass (e.g., dual-energy X-ray absorptiometry).

## Conclusions

The present study is the first to show the mediating role of food addiction across the relationships between each subdomain of childhood trauma and overweight and obesity in young adults. It is currently not known what other socioeconomic and psychological factors may contribute to these relationship. Emotional dysregulation has been associated with food addiction [44], and may be a contributor to disordered eating, particularly as a result of emotional abuse [32]. Future research may wish to examine the mediating role of emotional dysregulation in the relationship between childhood trauma and food addiction, and how this may differ across each subscale of childhood trauma. Mood disorders, such as depression, have also been associated with food addiction [45]. Future research should therefore explore how depression, anxiety, and post-traumatic stress may develop in response to childhood trauma and exacerbate the development of overweight and obesity among adolescents and young adults.

When attempting to understand the lifestyle factors associated with the growing global obesity pandemic, most research continues to focus on the role of diet and physical activity [46] However, many individuals struggle to initiate and maintain long-term weight loss with lifestyle interventions alone [3, 4]. The findings from this study show that people living with overweight or obesity may have experienced childhood trauma, which significantly increases their risk of maladaptive coping mechanisms (i.e., food addiction). Given that trauma and stress also adversely affects metabolism [47], these factors in combination can lead to the development of overweight and obesity. Thus, assessing and supporting people with underlying factors that increase likelihood of overweight or obesity, such as childhood trauma, and the associated mental health concerns, appears warranted, given that interventions focused solely on individual lifestyle behaviour change are unlikely to lead to weight loss maintenance and improved overall health. This study suggests that diverse approaches to care, where access to counsellors or clinical psychologists are available and part of multi-disciplinary team of health care professionals to support their goals to improve their health would be beneficial. In addition, due to the significant impact of childhood trauma in the development of food addiction, leading to overweight or obesity, early interventions supporting those who have experienced childhood trauma, and programs which aim to support vulnerable families to prevent trauma from occurring, are also essential and may contribute to the prevention of overweight and obesity.

### What is already known on this subject?

Evidence suggests that psychosocial risk factors, such as traumatic experiences during childhood, may increase the risk of developing obesity during adulthood. However, while the evidence suggests that childhood trauma is associated with obesity, the underlying mechanisms involved in this relationship are not well understood.

### What does this study add?

This is the first study to show that food addiction mediates the relationship between childhood trauma and overweight/obesity in young adults. These findings suggest many young adults who have experienced traumatic events during childhood, likely develop a food addiction as a maladaptive coping mechanism which leads to the development of overweight or obesity during early adulthood.

## Data Availability

The datasets generated during and/or analysed during the current study are available from the corresponding author on reasonable request.
